# Ream and Run Hemiarthroplasty Versus Total Shoulder Arthroplasty: A Comparison of Shoulder Treatments for Glenohumeral Arthritis

**DOI:** 10.7759/cureus.88813

**Published:** 2025-07-26

**Authors:** Leandra Roelker, Ali Ghasemi, Andrea Fabregas, Gene Shaffer, James Raphael

**Affiliations:** 1 Orthopaedics, Drexel University College of Medicine, Philadelphia, USA; 2 Orthopaedic Surgery, Einstein Healthcare Network, Philadelphia, USA; 3 Orthopaedics, Universidad Central del Caribe, Bayamón, USA

**Keywords:** glenohumeral arthritis, hemiarthroplasty, joint preservation, ream and run, shoulder arthroplasty

## Abstract

Glenohumeral arthritis is commonly managed by total shoulder arthroplasty (TSA), but risks of TSA failure due to its prosthetic glenoid component raise serious concerns, prompting some patients to turn to the ream and run technique (RnR) as an alternative procedure that avoids such complications. This systematic review and meta-analysis compared clinical outcomes of patients who underwent TSA or RnR treatment for glenohumeral arthritis. A total of 668 shoulders from a total of 666 patients were included, with 325 shoulders undergoing RnR and 343 shoulders undergoing TSA. Significant postoperative improvements in simple shoulder test (SST) score (RnR: 4.99, TSA: 4.51), American Shoulder and Elbow Surgeons (ASES) score (RnR: 40.50, TSA: 40.22), external rotation (RnR: 21.22º, TSA: 19.72º), and forward elevation (RnR: 24.75º, TSA: 40.50º) were found in both cohorts. A significant reduction in visual analog scale (VAS) pain score (RnR: -4.08, TSA: 3.93) was also found in both cohorts. Meta-analysis demonstrated no statistically significant difference between treatments across every outcome measure of the study. Both RnR and TSA techniques significantly improve pain and increase functionality and mobility in patients with glenohumeral arthritis. These findings have significant implications for clinical practice moving forward, as they offer clinicians and patients an additional treatment option with comparable outcomes.

## Introduction and background

Total shoulder arthroplasty (TSA) currently remains the preferred surgical method for treating glenohumeral arthritis when rotator cuff function remains intact, especially in the elderly [1]. TSA involves replacing both the humeral head and the glenoid surface with prosthetic implants to restore joint function. However, literature shows that although the procedure improves shoulder pain and function early on, patients risk five-year revision rates of at least 20% and lifelong activity limitations to prevent glenoid component loosening [2-4]. Particularly at risk are those highly active and/or younger, both factors which amplify the likelihood of progressive glenoid component wear, consequent osteolysis, and increased glenoid bone loss - a frequent postoperative complication and leading cause for TSA revision [5-7]. Worse still, with enough bone loss or scarring, surgical revision becomes increasingly unsuccessful [8].

**Figure 1:**
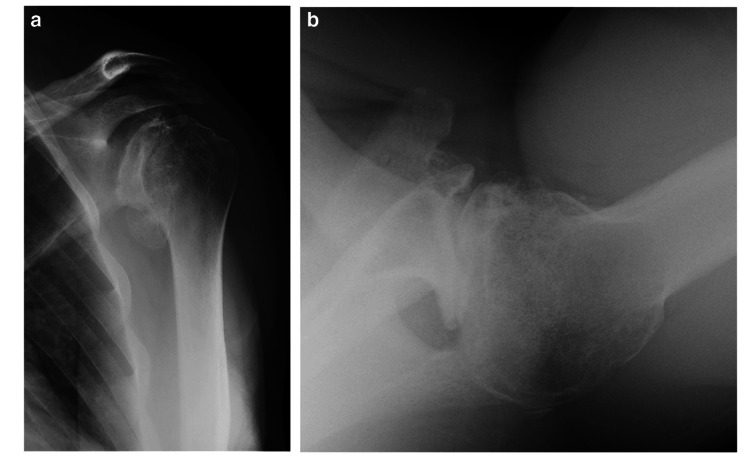
Preoperative Radiographs Demonstrating Glenohumeral Arthritis with Type B2 Glenoid Deformity Preoperative anteroposterior (AP) and axillary radiographs showing severe degenerative joint disease and a type B2 glenoid with biconcavity and posterior decentering of the humeral head. Used with permission from Matsen et al., International Orthopaedics (2019).

**Figure 2:**
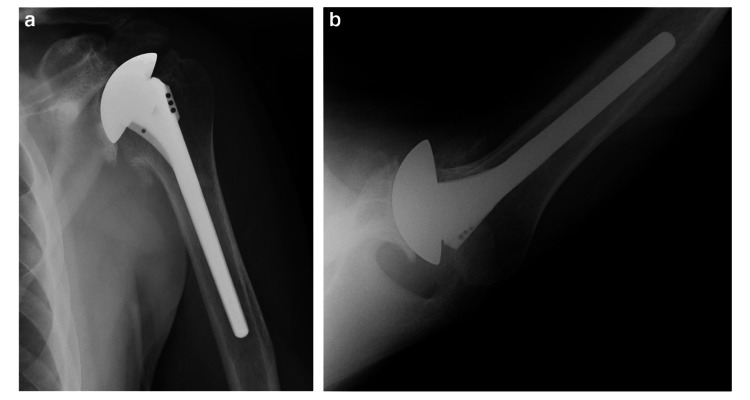
Postoperative Radiographs Following Ream-and-Run Humeral Hemiarthroplasty Postoperative anteroposterior (AP) and axillary radiographs showing centering of an anteriorly eccentric humeral head in the reamed glenoid, and fixation of a smooth-stemmed humeral component with impaction autografting. Used with permission from Matsen et al., International Orthopaedics (2019).

As a result, the ream and run procedure (RnR) pioneered by Matsen and Lippitt in the 2000s offers a potentially more suitable surgical alternative for patients in this demographic [9,10]. In contrast, RnR replaces only the humeral head and reshapes the glenoid bone without inserting a glenoid implant. The procedure combines a stemmed hemiarthroplasty with concentric glenoid reaming; most importantly, this modified hemiarthroplasty lacks the glenoid implant responsible for a major pitfall of glenohumeral arthritis management by TSA [8,11,12]. Instead, a humeral head prosthesis directly contacts the glenoid, reamed for durable articulation without implant [13]. Fibrocartilage regeneration is also triggered at the ream site and molded by glenohumeral motion. To mold the fibrocartilage appropriately, rehabilitation is begun immediately after surgery until healing is complete (Figures 1, 2) [10]. 

RnR also eliminates the activity limitations associated with glenoid preservation, but the procedure has concerns of its own: little research to date documents harmonious results. While some humeral hemiarthroplasty studies report favorable outcome improvement or procedural efficacy among patients who underwent RnR, others note increased pain, complications, and revision rates [8,14-19]. Postoperative stiffness is one such complication; younger age again raises the risk of both developing and requiring repeat procedures to treat [20,21]. The number of prior surgical procedures is also directly proportional to one’s likelihood of needing repeat procedures. A 2012 study suggests the ideal candidate for RnR may be a male patient with moderately good preoperative shoulder function and no prior shoulder surgeries [22]. A more recent study found parallel results, noting successful outcomes associated with male sex and lower preoperative simple shoulder test (SST) scores as well as more frequent reoperation in younger patients [23]. This systematic review aimed to aggregate such data to comprehensively compare outcomes of the TSA and RnR procedures and determine the ideal surgical treatment approach for patients with glenohumeral arthritis. 

## Review

Methods

Study Selection

The systematic review followed the Preferred Reporting Items for Systematic Reviews and Meta-Analyses (PRISMA) guidelines. Two authors conducted a literature search using two databases: PubMed and Scopus. The search terms included (ream and run) AND [(shoulder arthroplasty) OR (total shoulder)] to identify relevant articles. Filters were applied to include only studies involving human subjects and published in English. After the initial search, articles were manually selected for full-text review based on study titles and abstracts. Two reviewers independently screened all titles, abstracts, and full-text articles.

The analyzed data included 668 shoulders, collected from three articles. To be included in the review, studies had to directly compare the postoperative outcomes of RnR to TSA and provide complete data, including means, standard deviation/range, sample sizes, and patient demographics. Articles with overlapping cohorts and non-clinical publications were excluded. The methodological quality of the included studies was assessed using the modified Coleman Methodology Score (MCMS) and level of evidence.

An updated literature search was conducted in May 2025 using the same databases and search strategy. No additional comparative studies meeting the inclusion criteria were identified.

Data Extraction and Statistical Analysis

Demographic data, follow-up periods, revision rates, and comparable clinical outcome measures were extracted from each study. The mean and standard deviation (SD) of pre- and postoperative scores for the SST, American Shoulder and Elbow Surgeons (ASES) score, visual analog scale (VAS) for pain, and range of motion in active forward elevation and external rotation were aggregated from all reported clinical outcomes. To estimate the effect size of the treatment methods on the study outcomes, the mean difference (MD) and 95% confidence interval (CI) were calculated. The MD was determined by calculating the difference between the postoperative and preoperative scores. The heterogeneity in the results of the studies included in the meta-analysis was assessed using the Chi-square test, which determined the type of model (fixed or random). Additionally, the meta-regression method was employed to evaluate the difference in the MD of the scores for each of the indicators studied between the two treatment methods. All statistical analyses were conducted using Stata statistical software (version 15.0, Stata Corp, College Station, TX) with a significance level of less than 0.05.

Results

**Figure 3:**
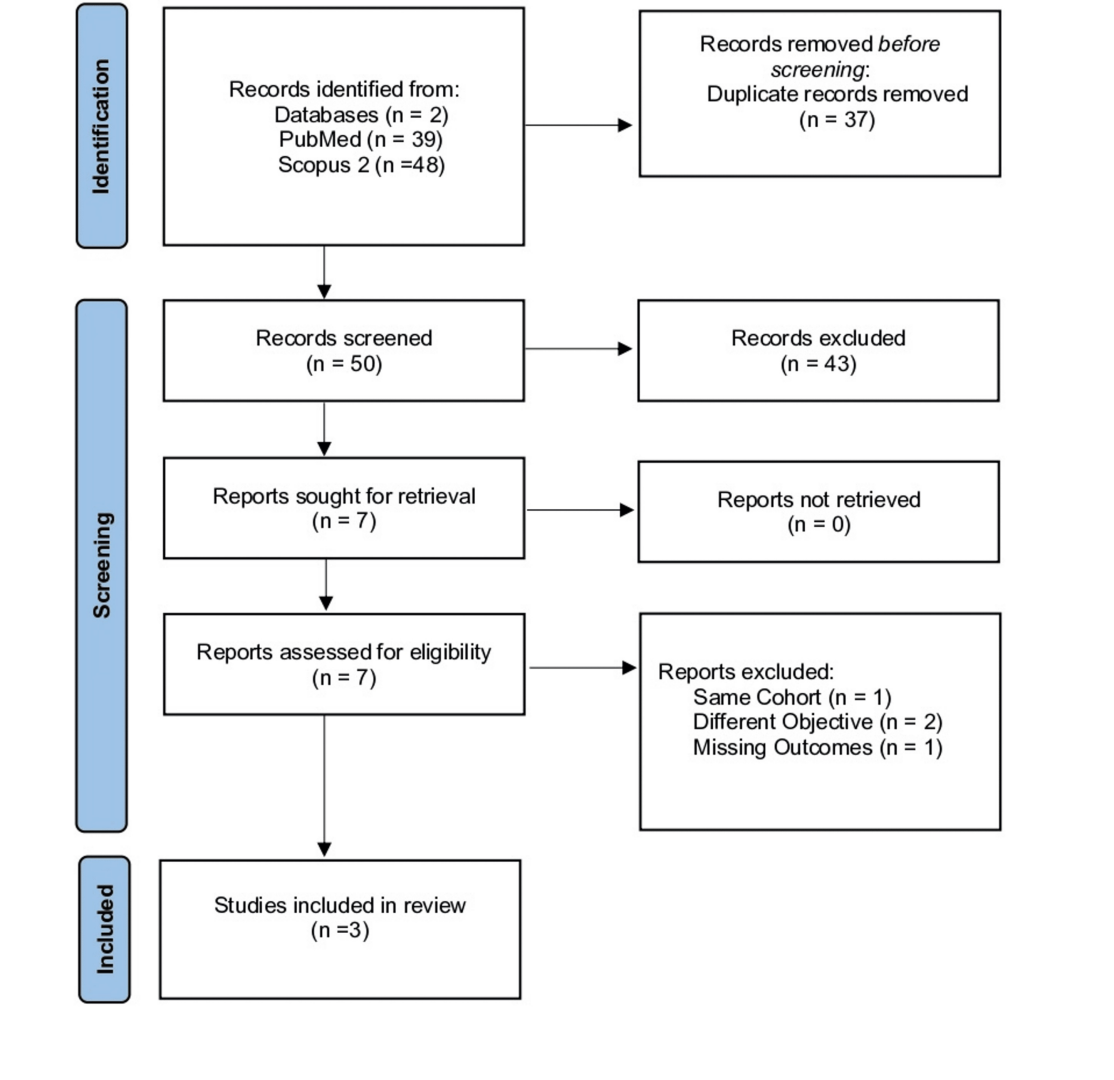
PRISMA Flow Diagram

Following a thorough review by two independent reviewers, three publications met the inclusion criteria (Figure 3) [15,24,25]. All three studies were cohort studies that used prospectively collected data to compare an RnR cohort to a TSA cohort, with an average follow-up of two years (level of evidence: III). The mean MCMS was 55, ranging from 54 to 57. A summary of study design, quality scores, and noted limitations is provided in Table 1.

**Table 1:** Summary of Study Quality and Bias Assessment Based on Modified Coleman Methodology Score (MCMS)

Study	Study Design	MCMS Score	Level of Evidence	Noted Limitations
Matsen et al. [15]	Retrospective Study	70	III	Non randomized design.
Levins et al. [24]	Retrospective Study	55	III	Single surgeon series with male only participants.
Virk et al. [25]	Retrospective Study	65	III	Small sample size.

Each article provided details on patient selection and surgical technique. All RnR procedures used a standard deltopectoral approach. Different techniques were employed to handle the subscapularis: one study used a lesser tuberosity osteotomy, another used a combined tenotomy-peel technique, and the third used a subperiosteal peel-back technique [15, 24, 25]. Glenoid reaming was performed using a reamer two mm larger in diameter than the humeral head prosthetic, following the Matsen RnR technique [12,15,24,25]. All studies described postoperative rehabilitation starting from day zero with passive range of motion exercises.

Patient Demographics

In total, this review included 668 shoulders (RnR: 325, TSA: 343) from 666 patients (Table 2). The mean age for the RnR group was 57.74±1.03 years; the mean age for the TSA group was 65.14±4.14 years. The overall mean follow-up time was 1.96±1 years. Patient characteristics varied across the publications, but all studies reported the percentage of patients who underwent subsequent procedures. The RnR cohort had a revision rate over two and a half times greater than the TSA cohort (RnR: 11.7%, TSA: 4.4%). Male to female ratios were also reported (RnR: 13.04, TSA: 1.27), as well as body mass index (BMI) mean RnR: 29.5, mean TSA: 30.4, percentage of narcotic use (RnR: 14.2%, TSA: 22.2%), percentage of active tobacco use at the time of surgery (RnR: 5.9%, TSA: 5.3%), and percentages of affected shoulders with prior surgery (RnR: 21.0%, TSA: 25.8%).

**Table 2:** Pooled Demographic Data for RnR and TSA Outcomes TSA: total shoulder arthroplasty, RnR: ream and run technique, BMI: body mass index.

Table 2. Pooled demographic data for RnR and TSA outcomes.					N (shoulders)		Mean Age		Mean Follow-up (years)		Male/Female		BMI		Active Tobacco Use		Narcotic Use		Prior Surgery		Subsequent Procedures	
Author	Year	Journal	Design	LOE	RnR	TSA	RnR	TSA	RnR	TSA	RnR	TSA	RnR	TSA	RnR	TSA	RnR	TSA	RnR	TSA	RnR	TSA
Virk et al. [25]	2018	Orthopedics	Cohort	III	23*	23	54 + 7.1	53 + 7.2	3.1 + 1.0	3.8 + 1.3	19/2	21/2	28.5 + 4.3	30.9 + 4.8	3	2	1	0	9	10	1	0
Matsen et al. [15]	2019	IO	Cohort	III	263	281	58 ± 9	67 ± 10	1.5 - 2.5	1.5 - 2.5	242/21	132/149	28.9 + 4.7	30.1 + 6.4	14	14	43	73	-	-	34	13
Levins et al. [24]	2023	JBJS	Cohort	III	39	39	58.2 + 8.8	58.9 + 5.5	4.2 + 1.8	4.5 + 2.7	39/0	39/0	31.0 + 6.0	30.2 + 4.7	-	-	2	3	4	6	3	2

Patient-Reported Outcome Measures

**Figure 4:**
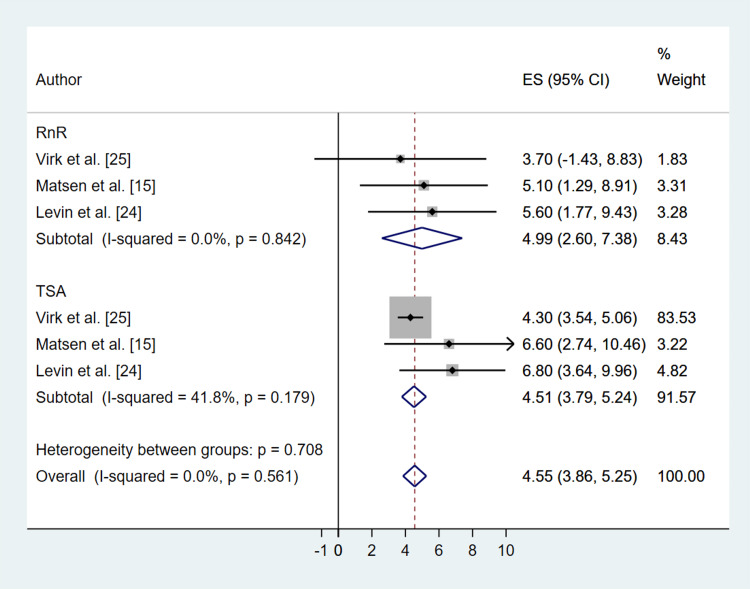
Comparative Forest Plot of Pre- Versus Post-Operation Simple Shoulder Test Score

**Figure 5:**
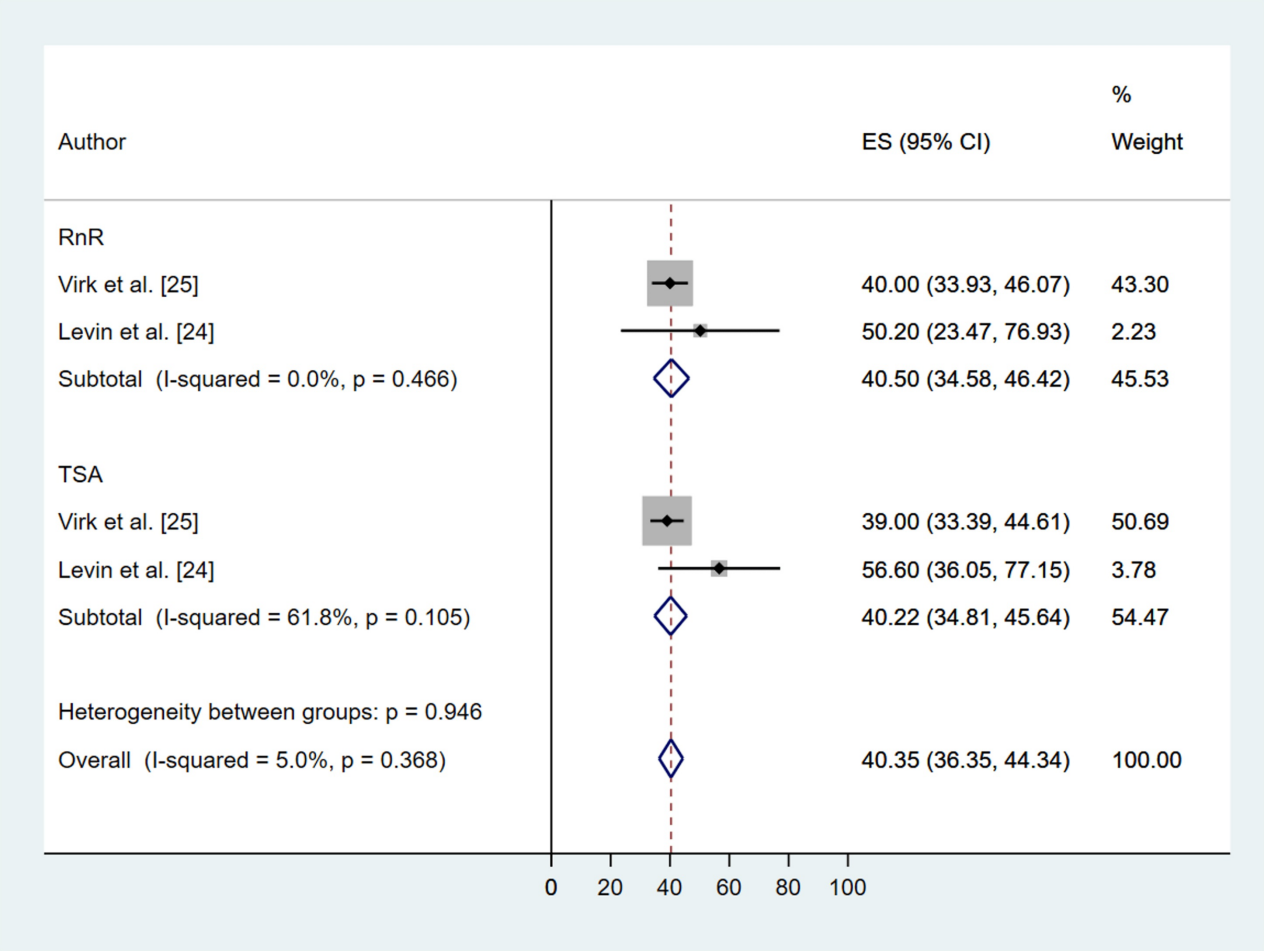
Comparative Forest Plot of Pre- Versus Post-Operation American Shoulder and Elbow Surgeons Score

**Figure 6:**
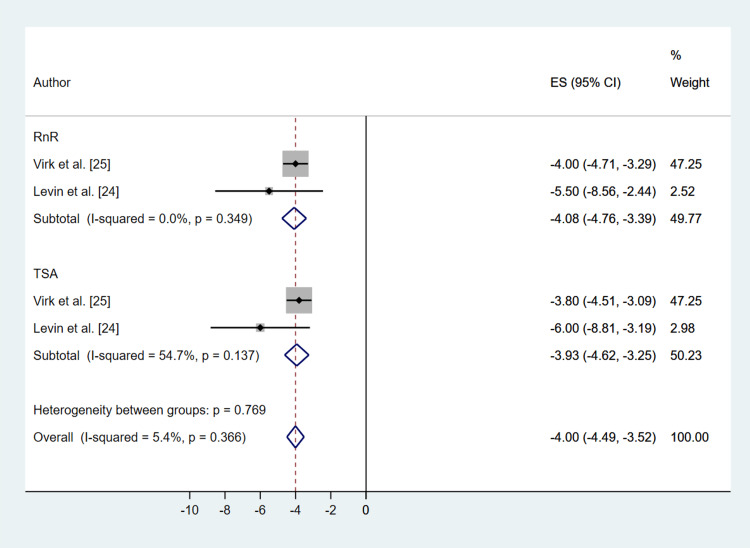
Comparative Forest Plot of Pre- Versus Post-Operation Visual Analog Scale Pain Score

All studies used validated patient-reported outcome measures (Table 3). The Simple Shoulder Test (SST) scores showed significant improvement in both the RnR (MD: 4.99; 95% CI: 2.60, 7.38; P ≤ 0.001) and TSA (MD: 4.51; 95% CI: 3.79, 5.24; P ≤ 0.001) groups, with no significant difference between the two treatment methods (P = 0.995) (Figure 4). ASES scores also significantly improved in both groups (RnR (MD: 40.50; 95% CI: 34.58, 46.42; P ≤ 0.001), TSA (MD: 40.22; 95% CI: 36.35, 44.34; P ≤ 0.001)), with no significant difference between treatments (P = 0.881) (Figure 5). Visual Analog Scale (VAS) pain scores decreased in both groups (RnR (MD: -4.08; 95% CI: -4.76, -3.39; P ≤ 0.001), TSA (MD: -3.93; 95% CI: -4.62, -3.25; P ≤ 0.001)), again with no significant difference between treatments (P = 0. 993) (Figure 6). Heterogeneity for all patient-reported outcomes was assessed using the Chi-square test, and forest plots (Figures 4-6) demonstrated low variability between studies, with no statistically significant heterogeneity detected. 

**Table 3:** Patient-Reported Outcome Measures SEM: standard error of mean

Table 3. Patient-reported outcome measures.			Simple Shoulder Test Score		American Shoulder and Elbow Surgeons Score		Visual Analog Scale Pain Score		Active forward elevation (º)		External Rotation (º)	
Author	Cohort	N	Pre Mean+SEM	Post Mean+SEM	Pre Mean+SEM	Post Mean+SEM	Pre Mean+SEM	Post Mean+SEM	Pre Mean+SEM	Post Mean+SEM	Pre Mean+SEM	Post Mean+SEM
Virk et al. [25]	RnR	23	6.2+0.6	9.9+3.0	45 + 4	85 + 4	5.4 + 0.5	1.4 + 0.4	115 + 6	139 + 6	26 + 3	47 + 4
	TSA	23	5.6+0.5	9.9+0.5	46 + 4	85 + 3	4.7 + 0.5	0.9 + 0.3	113 + 9	154 + 5	39 + 5	58 + 3
Matsen et al. [15]	RnR	263	4.9 ± 2.4	10.0 ± 2.6	-	-	-	-	-	-	-	-
	TSA	281	2.9 ± 2.3	9.5 ± 2.7	-	-	-	-	-	-	-	-
Levins et al. [24]	RnR	39	4.7 + 2.7	10.3 ± 2.2	34.8 ± 15.2	85.0 ± 18.9	7.0 ± 1.9	1.5 ± 2.1	109.0 ± 24.0	144.5 ± 12.8	12.2 ± 22.6	40.8 ± 12.5
	TSA	39	4.1 + 2.2	10.9 ± 1.9	33.3 + 14.1	89.9 ± 12.8	7.1 ± 1.9	1.1 ± 1.8	106.8 ± 26.1	142.8 ± 12.2	12.1 ± 21.8	45.2 ± 14.9

Radiographic Outcomes

**Figure 7:**
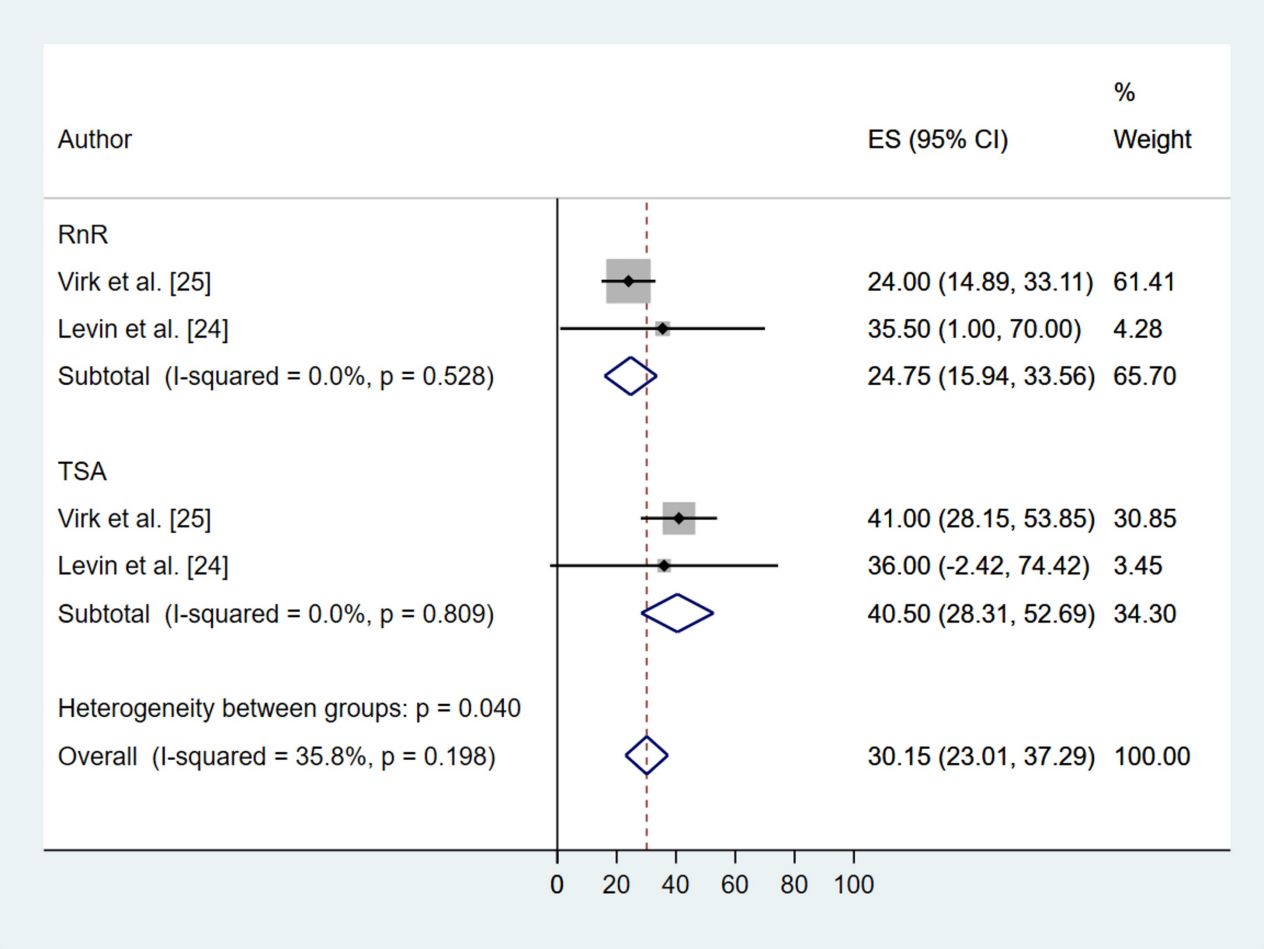
Comparative Forest Plot of Pre- Versus Post-Operation Active Forward Elevation

**Figure 8:**
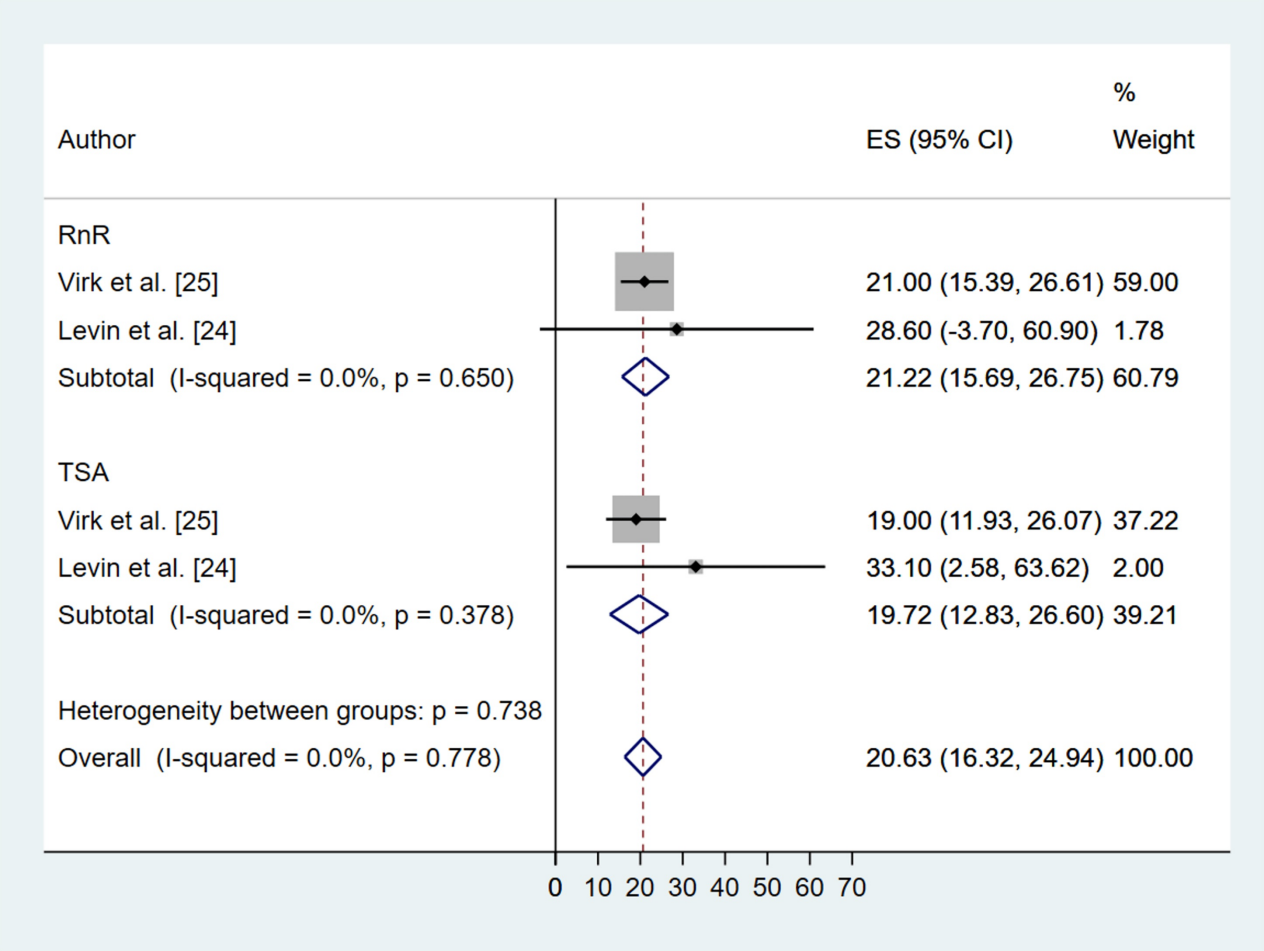
Comparative Forest Plot of Pre- Versus Post-Operation External Rotation

Radiographic outcomes were reported for active forward elevation and external rotation (Table 2). Both treatment interventions significantly increased the degree of forward elevation [RnR (MD: 24.75; 95% CI: 15.94, 33.56; P ≤ 0.001), TSA (MD: 40.50; 95% CI: 28.31, 52.69; P ≤ 0.001)] (Figure 7), and external rotation [(RnR (MD: 21.22; 95% CI: 15.69, 26.75; P ≤ 0.001), TSA (MD: 19.72; 95% CI: 12.83, 26.60; P ≤ 0.001)] (Figure 8), with no significant difference between the two treatments and P = 0.177 and 0.770 for forward elevation and external rotation, respectively.

Discussion

Clinical Implications & Decision Making

Primarily, this study finds that both TSA and RnR effectively increase function and relieve pain in glenohumeral arthritis patients. Moreover, our meta-analyses show no significant difference between methods in improving every examined outcome measure (SST score, ASES score, VAS pain score, active forward elevation, and external rotation) - demonstrating the success of RnR as a treatment option for glenohumeral arthritis, given that a patient meets the appropriate criteria for the procedure.

Getz et al. and Somerson et al. previously demonstrated that in the right demographic, RnR is capable of improving function and reducing pain for glenohumeral arthritis patients with an intact rotator cuff [14,17]. Somerson et al. later confirmed that these results were sustained at a mid-term follow-up of ten years, one of the longest follow-up terms on the RnR procedure to date [16].

Given the higher revision rate observed in the RnR group, even with comparable functional outcomes, careful consideration should be given when selecting younger or more active patients, especially in the presence of other risk factors like tobacco or narcotic use. Differences in age and sex may reflect inherent selection bias, which can limit the broader applicability of these results.

Surgical Technique Variability

Qualifying criteria for RnR as used by the aforementioned studies as well as those of this review are delineated by Matsen: 1) strongly motivated patients who are 2) physically, mentally and emotionally capable of completing a daily rehabilitation program that may last up to two years and who are 3) likely capable of forming fibrocartilage on the newly reamed glenoid are appropriate candidates for RnR [10,15,24,25]. Subsequently, Levins et al., Matsen et al., and Virk et al. consistently reported that younger males with high pre-surgical optimism and less frequent histories of narcotic or tobacco use had equivalent patient-reported outcomes following RnR as patients who underwent TSA [15,24,25].

Unfortunately, such results suggest patients with a history of depression, substance use, or comorbidities hindering dedicated postoperative rehabilitation are instead better candidates for TSA. Patients with systemic illness or on medications impairing cartilage growth also risk decreased clinical outcomes with an RnR procedure and should consider alternative surgical treatment options [10].

Statistical Considerations

As the current body of evidence stands, it seems the patients who stand to benefit most from the RnR procedure are nearly identical to those at greatest risk for potential long-term complications of TSA. Although largely successful in older patients, younger patients with eccentric posterior glenoid wear experience varied results following the latter procedure, prompting considerable emphasis on the fact that operative management in the unique group of glenohumeral arthritis patients less than 55 years old must consider a treatment method that preserves joint longevity and can withstand greater load-bearing and high activity demand [20,24,26-30]. In light of this review, RnR may be the ideal fit for this demographic, provided appropriate surgeon expertise. Although statistical differences between groups were not observed, the magnitude of mean differences in measures such as VAS pain and forward elevation may still carry clinical relevance depending on patient-specific goals and functional expectations.

Additionally, as noted by Matsen and Somerson, outcomes following RnR may depend on the patient’s willingness to adhere to a rehabilitation program and maintain high motivation throughout recovery. Also, the importance of early identification of patients less likely to succeed with RnR based on psychological readiness or systemic comorbidities was emphasized in these studies.

Limitations

Because little literature exists comparing the RnR and TSA procedures, the greatest inevitable limitations of this review are its selection bias and relatively small, retrospective cohorts. With a majority of the data coming from a single-center study and a maximum follow-up of just 4.2 years, large prospective studies and studies reporting mid- to long-term follow-up for the RnR procedure in particular are still much needed.

Additionally, the included RnR cohorts differed in their subscapularis management: tenotomy, peel-back, and osteotomy, which could have impacted outcomes but were not examined independently. The demographic differences observed between cohorts, especially age and sex, introduce potential bias, as younger and predominantly male patients were more likely to undergo RnR. This imbalance was not explored using subgroup analysis and should be considered when interpreting the results. Follow-up periods were also relatively short in two of the studies, limiting assessment of long-term results like implant survival or glenoid wear. Although the RnR cohort had more than twice the revision rate as TSA, this difference wasn’t explored in detail and likely warrants further study. Other factors, such as the financial cost of each procedure and the demands of prolonged rehabilitation, weren’t addressed, despite their importance in clinical decision-making. Finally, most of the reviewed data came from high-volume academic centers, so the findings might not generalize well to surgeons or patients outside those settings.

## Conclusions

In conclusion, this study provides evidence that both the RnR and TSA treatment methods are effective in improving patient-reported outcome measures, reducing pain, and enhancing shoulder mobility in patients with glenohumeral arthritis. These findings have significant implications for clinical practice, as they offer clinicians and patients options for shoulder treatment with comparable outcomes.

However, differences in surgical technique, particularly in subscapularis management during RnR, could have potentially affected our outcomes and remain an area for further investigation. Long-term follow-up data are essential to determine the durability of pain relief, functional improvement, and implant survivorship over time, particularly given the higher revision rate seen in RnR patients in short-term follow-up.

Selection bias, especially related to age, sex, and preoperative shoulder function, could have contributed to the observed results and should be addressed in future matched-cohort or randomized studies. Though our statistical analysis revealed no significant heterogeneity between outcomes, the limited number of studies restricts the power to fully detect subtle effect size differences, highlighting the need for larger, multi-center analyses. Additionally, future research should assess not just clinical outcomes but also cost-effectiveness and patient adherence to postoperative rehabilitation, both of which can strongly influence the real-world success of the RnR procedure.
